# Range‐wide population genomics of the Mexican fruit fly: Toward development of pathway analysis tools

**DOI:** 10.1111/eva.12824

**Published:** 2019-06-13

**Authors:** Julian R. Dupuis, Raul Ruiz‐Arce, Norman B. Barr, Donald B. Thomas, Scott M. Geib

**Affiliations:** ^1^ U.S. Department of Agriculture‐Agricultural Research Service Daniel K. Inouye U.S. Pacific Basin Agricultural Research Center Hilo Hawaii; ^2^ Department of Plant and Environmental Protection Sciences University of Hawai’i at Mānoa Honolulu Hawaii; ^3^ U.S. Department of Agriculture‐Animal and Plant Health Inspection Service, Plant Protection & Quarantine, Science and Technology Mission Laboratory Edinburg Texas; ^4^ U.S. Department of Agriculture‐Agricultural Research Service Cattle Fever Tick Research Laboratory Edinburg Texas

**Keywords:** agricultural pest, *Anastrepha*, ddRAD, Fluidigm, pathway analysis, source determination, Tephritidae

## Abstract

Recurrently invading pests provide unique challenges for pest management, but also present opportunities to utilize genomics to understand invasion dynamics and inform regulatory management through pathway analysis. In the southern United States, the Mexican fruit fly *Anastrepha ludens* is such a pest, and its incursions into Texas and California represent major threats to the agricultural systems of those regions. We developed a draft genome assembly for *A. ludens*, conducted range‐wide population genomics using restriction site‐associated DNA sequencing, and then developed and demonstrated a panel of highly differentiated diagnostic SNPs for source determination of intercepted flies in this system. Using 2,081 genomewide SNPs, we identified four populations across the range of *A. ludens*, corresponding to western Mexico, eastern Mexico/Texas, Guatemala/Belize/Honduras, and Costa Rica/Panama, with some intergradation present between clusters, particularly in Central America. From this population genomics framework, we developed a diagnostic panel of 28 highly differentiated SNPs that were able to recreate the genomewide population structure in this species. We demonstrated this panel on a set of test specimens, including specimens intercepted as part of regular trapping surveillance in Texas and California, and we were able to predict populations of origin for these specimens. This methodology presents a highly applied use of genomic techniques and can be implemented in any group of recurrently invading pests.

## 1 INTRODUCTION

In the current era of unprecedented global movement and trade, biological invasions are an ever‐growing threat to both ecosystems and economies (Oerke, 2005; Paini et al., 2016; Pimentel, Zuniga, & Morrison, 2005; Pyšek & Richardson, 2010). Invasive insects can be particularly detrimental due to their high incidence of anthropogenic movement (Kenis et al., 2009; Kiritani & Yamamura, 2003; Sakai et al., 2001), often becoming serious pests of agriculture and forestry (Aukema et al., 2010; Dukes et al., 2009; Paini et al., 2016; Pimentel et al., 2005). However, perhaps due in part to humanity's experience with and long history of managing insect pests (Dales, 1996; Kogan, 1998), eradication of invasive insect populations from non‐native ranges can be successful (Liebhold et al., 2016; Myers, Simberloff, Kuris, & Carey, 2000). Although control measures are taxon‐specific (Liebhold et al., 2016), this is most often achieved through combinations of mass trapping, insecticidal sprays or baits, microbial pesticides, host destruction, and sterile insect technique (SIT, see below) (Dyck, Hendrichs, & Robinson, 2005; Kogan, 1998; Liebhold et al., 2016). Prompt detection and the geographic scale of invasion are often some of the most important factors dictating successful eradication, as early detections of small or confined invasions generally have a greater probability of successful eradication (Myers et al., 2000; Suckling et al., 2016). In cases where eradication can be accomplished, but recurrent invasions occur by the same species, constant surveillance along with both preventative and reactionary management is required to avoid establishment (Liebhold et al., 2016; Myers et al., 2000). These recurrently invading pests present unique challenges for pest management, but also unparalleled opportunities to understand the pathways and mechanisms of biological invasions (Barr, Ruiz‐Arce, & Armstrong, 2014).

Population genetics provides unique evolutionary perspective on invasive systems (Lee, 2002; Mooney & Cleland, 2001). Population genetic tools are useful for identifying and characterizing invasive populations and their demographics (Estoup, Beaumont, Sennedot, Moritz, & Cornuet, 2004; Genton, Shykoff, & Giraud, 2005; Tsutsui & Case, 2001), reconciling invasion histories (Abdelkrim, Pascal, & Samadi, 2005; Estoup & Guillemaud, 2010; Mori, Davis, & Evenden, 2016; Zhang, Edwards, Kang, & Fuller, 2014), and evaluating the success of control efforts (Abdelkrim et al., 2005; Robertson & Gemmell, 2004). In the case of recurrently invading species, population genetics offers the ability to trace individual introductions to their geographic source, which can be important for identifying patterns of invasion and thus potential weaknesses in established biosecurity systems. Such “assignment tests” (Paetkau, Calvert, Stirling, & Strobeck, 1995; Taylor, Beacham, & Kaeriyama, 1994) are widely used in population and conservation genetics (Cornuet, Piry, Luikart, Estoup, & Solignac, 1999; Manel, Gaggiotti, & Waples, 2005). For example, microsatellite markers have been used to assign black bears to their natal range (Puckett & Eggert, 2016) and identify the geographic origin of poached elephant ivory (Wasser et al., 2015, 2004), and panels of single nucleotide polymorphisms (SNPs) have regularly been used to identify populations and hatchery stocks of salmon (Larson, Seeb, Everett, et al., 2014; Storer et al., 2012; Templin, Seeb, Jasper, Barclay, & Seeb, 2011). Such intraspecific diagnostic markers have also been suggested for use in forestry and agriculture systems (Dupuis, Sim, et al., 2017; Picq et al., 2018), but have not been extensively demonstrated for recurrently invading insect pests in these settings. The success of these approaches depends heavily on many factors, including the type of molecular marker used, the relative differentiation and the corresponding structure between populations, population changes over time, the presence of a reference collection representing the geographic range, and in the case of panels of highly differentiated SNPs (discussed below), the manner in which the most diagnostic markers are selected (Anderson, 2010; Benestan et al., 2016, 2015; Larson, Seeb, Pascal, Templin, & Seeb, 2014; Negrini et al., 2009; Puckett & Eggert, 2016; Storer et al., 2012).

Genomic approaches offer increasing accessibility to large datasets of SNPs, which can greatly increase the power of population genetic methods generally (Luikart, England, Tallmon, Jordan, & Taberlet, 2003; Rašić, Filipović, Weeks, & Hoffmann, 2014) and assignment tests specifically (Chown et al., 2015; Puckett & Eggert, 2016). Given this increased availability, a common approach for developing a panel of diagnostic molecular markers for population assignment is to generate a genomewide SNP dataset for populations of interest (thousands of SNPs) and then select a smaller panel of highly informative SNPs (dozens to hundreds of SNPs) that could be genotyped in a cost‐effective manner (Dupuis, Sim, et al., 2017; Larson, Seeb, Pascal, et al., 2014; Puckett & Eggert, 2016). Highly informative markers can be chosen using different criteria (*F*_ST_‐based, contribution to principal components, etc.), which can potentially affect the relative success of assignment tests (Negrini et al., 2009; Storer et al., 2012). Once a small set of highly informative markers is chosen, fast and cost‐effective genotyping, such as with specialized SNP genotyping assays (Templin et al., 2011) or high‐resolution melt analyses (Storer et al., 2012), can be validated to increase the general accessibility of the tool in a diagnostic context. Despite this attractive workflow, few studies in the realms of agriculture and forestry pests go as far as to develop and validate a fast and cost‐effective genotyping assay for such diagnostic capability.

Fruit flies in the family Tephritidae are an ideal group of recurrently invading pests that are in need of such diagnostic molecular tools for source determination (Barr et al., 2014). Individual tephritid species can attack hundreds of species of hosts, including many commercially produced fruits and vegetables, and several species are recognized as some of the most destructive and economically damaging agricultural insect pests in the world (e.g., the oriental fruit fly *Bactrocera dorsalis* (Hendel) and Mediterranean fruit fly *Ceratitis capitata* (Wiedemann)) (Aluja & Mangan, 2008; Bateman, 1972; Papadopoulos, Plant, & Carey, 2013). Adult females oviposit in ripening fruit (and various other plant tissues depending on the species) and larval feeding and subsequent microbial growth can cause extensive fruit decay and fruit drop. While this direct damage to produce can have economic effects, additional economic impact results from global movement of these flies, their establishment in non‐native ranges, and subsequent pest management efforts, including eradication (White & Elson‐Harris, 1992). Given the widespread nature of invasive tephritids, importing countries often have restrictions for movement of produce from fruit fly‐established areas (White & Elson‐Harris, 1992). “Fruit fly‐free” countries that have climates amenable to these pests undertake extensive surveillance (trapping grids/networks) and quarantine measures to detect invasive flies and prevent their establishment (Papadopoulos et al., 2013; Shelly, Epsky, Jang, Reyes‐Flores, & Vargas, 2014; White & Elson‐Harris, 1992). When flies are intercepted in these trapping networks, pest management response can include pesticide application and bait‐and‐kill mass trapping, destruction of potential host material (e.g., fruit stripping), and SIT, which is the mass‐rearing, sterilization (via irradiation), and release of flies (ideally, males only), which mate with wild, fertile flies leading to no reproductive output and population suppression (Dyck et al., 2005). Such eradications can cost in the range of $0.1–240 million USD per eradication campaign (Papadopoulos, 2014; Suckling et al., 2016), and it has been estimated that establishment of the Mediterranean fruit fly in California could have an economic impact of 1.4 billion USD in the first year alone (http://www.cdfa.ca.gov). Given the cost of eradication and establishment‐prevention measures and additional impacts of subsequent quarantines, regulatory agencies have high demand for population genetic tools for geographic source determination, both to respond to individual invasions and to identify trends in invasion route over time (Barr et al., 2014).

The Mexican fruit fly *Anastrepha ludens* (Loew) (Diptera: Tephritidae) is one of the most serious fruit fly pests in the tropical Americas (Norrbom & Foote, 1989) and is distributed from the far southern United States, throughout Mexico and Central America (Enkerlin, Garcia, & Lopez, 1989; Ruiz‐Arce, Owen, Thomas, Barr, & McPheron, 2015; Stone, 1942; White & Elson‐Harris, 1992). As with other tephritid pests, female *A. ludens* use their slender ovipositor to lay eggs under the skin of ripening fruit, and larval feeding and subsequent microbial growth can cause extensive fruit decay and fruit drop. The main commercial crops affected by *A. ludens* are citrus (Rutaceae: *Citrus* spp.) and mango (Anacardiaceae: *Mangifera indica* L.), although it has been recorded from >30 other fruits (Norrbom & Kim, 1988; White & Elson‐Harris, 1992). This species is of particular phytosanitary concern for the United States, as flies are routinely intercepted in several states in the southern United States (Ruiz‐Arce et al., 2015; Steck, 1998), and quarantines are commonly established to eradicate invasive populations in California and Texas (Ruiz‐Arce et al., 2015; USDA‐APHIS, 2010). Successful eradication of these populations is greatly facilitated by the use of SIT (Orozco, Dominguez, Reyes, Villasenor, & Gutierrez, 2002; Orozco, Meza, Zepeda, Solís, & Quintero‐Fong, 2013), and multiple rearing strains are maintained in both Guatemala and Texas by the United States Department of Agriculture. Three of these strains have been historically released as part of eradication efforts in south Texas: Willacy, Tapachula 7, and Family 10. Willacy is a wild‐type strain, recently established from fewer than five wild *A. ludens* collected in Willacy County, Texas (Conway, H. personal communication). In contrast, Tapachula 7 and Family 10 are genetic sexing strains, which both harbor a mutation for black pupae, which is made sex‐linked through a stable autosomal translocation with the Y chromosome (Rendón, personal communication; Orozco et al., 2013; Zepeda‐Cisneros et al., 2014). This results in males having a wild‐type brown pupal color, fixed in a heterozygous state, while females have a phenotypic black pupa and are homozygous recessive for this trait. Both strains were derived from the same original black pupae strain but have novel translocations to allow sex linkage of the phenotype (P. Rendón, *personal communication*).

Here, we present the first genomic evaluation of population structure across the full geographic range of *A. ludens*. We generate genomewide SNP markers with double‐digest restriction site‐associated DNA sequencing (ddRAD, Peterson, Weber, Kay, Fisher, & Hoekstra, 2012) and assemble a reference genome from fragment and mate‐pair libraries to facilitate SNP genotyping. We then mine the resulting population genomic dataset (2,081 filtered SNPs) for highly informative SNPs and develop a panel of diagnostic markers (28 SNPs) capable of geographic source determination for *A. ludens*. We implement and validate this diagnostic panel using rapid and low‐cost SNP genotyping assays and then demonstrate its utility in a real‐world test by genotyping a set of specimens of unknown origin, which includes flies and maggots intercepted as part of routine monitoring efforts in California and Texas from 2014–2016. This study provides both a framework for, and a demonstrated example of, development of robust diagnostic tools from genomic resources for source determination of invasive pests of phytosanitary concern.

## 2 MATERIALS AND METHODS

### 2.1 ddRAD specimen selection, DNA extraction, and library preparation

Wild *A. ludens* were collected from 1998 to 2006 and included adults caught in protein‐baited traps (generally Torula yeast hydrolysate or grape juice concentrate) and larvae extracted from host fruit material. Specimens were preserved in 95% ethanol or frozen at −80°C, and species identifications were based on morphology and conducted by DBT. The majority of our sampling are flies from the same traps that were previously investigated using sequencing methods by Ruiz‐Arce et al. (2015), and we also included specimens from the three rearing strains, Willacy, Tapachula 7, and Family 10. We homogenized whole flies using a 3.175 mm 18/10 stainless steel bearing in 200 µl tissue lysis buffer (Macherey–Nagel, Düren, Germany) with a GenoGrinder 2010 (Spex Sample Prep, Metuchen, NJ) in 96‐well format at a speed of 4.0 m/s for 20 s. We then incubated tissue homogenates overnight at 55°C with 25 µl proteinase K (23 mg/ml: Macherey–Nagel, Düren, Germany), before finishing the extraction with a KingFisher Flex‐96 automated extraction instrument (Thermo Scientific, Waltham, MA) and Mag‐Bind Tissue DNA KF Kits (Omega, BioTek, Norcross, GA) following manufacturer's recommendations. We conducted the optional RNase A treatment, eluted DNA into 100 µl Mag‐Bind elution buffer, and quantified the extractions using a SpectraMax M2 plate reader (Molecular Devices, Sunnyvale, CA) with a PicoGreen assay. Finally, we normalized DNA to 4 ng/µl in 44.5 µl dH_2_O using a Gilson PIPETMAX 268 (Gilson, Middleton, WI).

We followed the approach of Peterson et al. (2012) to prepare ddRAD libraries. We used ~178 ng of input DNA and the restriction enzymes NlaIII and MluCI for library preparation, and the initial ligation was performed with 48 unique barcoded adapters. We size‐selected subpools of these adapters with a Blue Pippin electrophoresis unit (Sage Science, Beverly, MA), using a 1.5% agarose gel cassette and the “narrow 450 bp” target size setting, and then conducted an additional PCR to add Illumina i7 barcodes to each subpool. Subpool libraries were purified using 1.5:1 polyethylene glycol with solid‐phase reversible immobilization beads: sample (DeAngelis, Wang, & Hawkins, 1995), and then quantified with a 2,100 Bioanalyzer (Agilent, Santa Clara, CA) using the high‐sensitivity DNA kit. Three final libraries were created by pooling cleaned subpool libraries at equal molar ratios (each final library contained 190 individuals and two negative control samples), and each final library was sequenced on a single lane of HiSeq 4,000 (Illumina, San Diego, CA) with 100‐bp single‐end sequencing.

### 2.2 Generating an *A. ludens* reference genome

To provide a reference assembly for mapping reads from the ddRAD data, we generated libraries that were optimized to be assembled using the ALLPATHS‐LG assembler (Gnerre et al., 2011). We used flies collected from the rearing colony maintained at the Petapa San Miguel Mexfly Mass Rearing Facility in Guatemala, collected from the Family 10 black pupae genetic sexing strain, and extracted DNA as for the ddRAD specimens. We constructed a 180‐bp insert Illumina TruSeq fragment library from 500 ng DNA from a single female individual, and an Illumina Nextera mate‐pair library targeting a 3‐kb insert size from a pool of sibling male individuals (eight individuals in total), to have sufficient DNA for mate‐pair library construction. The fragment and mate‐pair libraries were each sequenced on a lane of Illumina HiSeq4000 with 2 × 100 bp paired‐end sequencing and 2 × 50 bp paired‐end sequencing, respectively. We constructed a scaffold assembly from raw reads of both libraries using ALLPATHS‐LG (Gnerre et al., 2011). We performed k‐mer‐based error correction (using the ALLPATHS‐LG pipeline) to the fragment library and then ran the pipeline with default settings except for the addition of the “HAPLOIDIFY = TRUE” parameter.

### 2.3 ddRAD data processing

We used the *Stacks* pipeline v1.35 (Catchen, Amores, Hohenlohe, Cresko, & Postlethwait, 2011; Catchen, Hohenlohe, Bassham, Amores, & Cresko, 2013) to demultiplex, map to the ALLPATHS‐LG reference, and call SNPs from the ddRAD data. First, *process_radtags* was used to demultiplex raw sequencing files, remove reads with uncalled bases and low‐quality scores, and rescue those with errors in the cut‐site or barcode. The Burrows‐Wheeler Aligner v0.7.12 (Li & Durbin, 2009) was then used to map reads to the reference using the MEM algorithm (Li, 2013), and *Stacks*’ *ref_map.pl* was used to assemble loci and generate a catalog, and then match individuals to that catalog. A single mismatch between loci was allowed when building the catalog, and a minimum coverage of three reads was required to report a stack during the *pstacks* part of the pipeline. Finally, we used *populations* to generate final SNP datasets in vcf format, using a population map that assigned all individuals to a single population. For a locus to be included, we required it to be present in one population and in a minimum of 1% of individuals. We also used a minimum stack depth per individual per locus of six reads and wrote a single SNP per catalog locus. We then filtered the output of *populations* using VCFtools v0.1.14 (Danecek et al., 2011). First, we identified individuals with high missing data by calculating individual missingness with a per‐locus missing data filter of 50%; individuals with >50% missing data were excluded from subsequent filtering. We then removed loci with >20% missing data and a minor allele frequency <1% to create an input dataset for population genetic analyses. We used PGDSpider v2.1.0.3 (Lischer & Excoffier, 2012) for data conversion from vcf format into the various formats needed for downstream analyses.

### 2.4 Population genetic analyses

To assess broad patterns of population structure, we conducted individual‐based Bayesian clustering in STRUCTURE v2.3.4 (Pritchard, Stephens, & Donnelly, 2000), which assigns individuals to genetic clusters that maximize gametic and Hardy–Weinberg equilibria. We assessed *K* = 1–20 using 40 replicates of each *K*, with correlated allele frequencies (Falush, Stephens, & Pritchard, 2003), the admixture model, 50,000 Markov chain Monte Carlo replicates of burn‐in followed by 100,000 sampled replicates, and otherwise default parameters. As these analyses were aimed to determine population structure of wild populations, we excluded individuals from rearing strains in the main STRUCTURE analysis. However, we also ran identical sets of analyses including these rearing individuals, as well as sets of analyses using a location prior, and, following the recommendation of Wang (2016) for datasets with uneven sample sizes, with *α* = 1/*K* (with *K* = 5, based on preliminary analyses) and the alternative ancestry prior. For location prior analyses, and to avoid populations with low sample sizes, we grouped individuals by medium‐sized political regions (Texas, Central American countries individually, and states in Mexico, leading to 23 populations, Table S1). We averaged results across runs and calculated Ln Pr(*X|K*) (Pritchard et al., 2000), *ΔK* (Evanno, Regnaut, & Goudet, 2005), and the statistics proposed by Puechmaille (2016), using CLUMPAK (Kopelman, Mayzel, Jakobsson, Rosenberg, & Mayrose, 2015) and Structure Selector (Li & Liu, 2017). The Puechmaille statistics require a priori population groupings and a specified threshold value; for these parameters, we used the same population groupings as for the location prior STRUCTURE analysis and a threshold of 0.5 to account for high levels of admixture in the dataset (see Puechmaille, 2016), respectively. We considered all three statistics to determine the most likely value of *K*.

We also used discriminant analysis of principal components (DAPC, Jombart, Devillard, & Balloux, 2010) to assess population structure in the dataset. This multivariate method attempts to maximize between‐group and minimize within‐group variability in a set of predefined groups by first conducting a principal component analysis and then subjecting those principal components to a discriminant analysis (Jombart et al., 2010). We used the *adegenet* package v2.1.1 (Jombart, 2008) in R v3.4.4 (R Core Team, 2018) to conduct DAPC. The *find.clusters* function was used to estimate *K* (retaining all principal components), and *xvaldapc* was used to perform cross‐validation to assess the optimal number of principal components to retain in the discriminant analysis (considering a maximum value of 200 principal components). Like with STRUCTURE, we conducted DAPC with and without inclusion of the rearing individuals.

We calculated basic population genetic statistics (heterozygosity, inbreeding coefficient, gene diversity, etc.) and pairwise population differentiation using GenoDive v2.0b27 (Meirmans & Van Tienderen, 2004). For the latter, we used 10,000 permutations of the analysis of molecular variance (AMOVA) *F*_ST_ method (Excoffier, Smouse, & Quattro, 1992; Michalakis & Excoffier, 1996) and applied a Bonferroni correction to account for multiple comparisons. Finally, we used GENEPOP v1.2 (Raymond & Rousset, 1995; Rousset, 2008) to calculate gene diversity among individuals in a population (1–*Q*_inter_).

### 2.5 Diagnostic markers for source determination

We used a measure of locus‐specific genetic differentiation to select a subset of highly differentiated ddRAD loci to create a panel of diagnostic markers for *A. ludens*, which we then implemented with rhAmp SNP genotyping assays (Integrated DNA Technologies, Coralville, IA). We calculated Weir and Cockerham's *F*_ST_ (Weir & Cockerham, 1984) for each locus in the filtered population genetic dataset with VCFtools v0.1.14 (Danecek et al., 2011) and based population groupings on both country of origin and the main results of STRUCTURE (*K* = 5, excluding rearing individuals); this led to seven groups, corresponding to (a) western Mexico and (b) Texas/eastern Mexico, as differentiated by STRUCTURE, (c) Guatemala, (d) Belize, (e) Honduras, (f) Costa Rica, and (g) Panama. We then manually filtered the ddRAD loci to a subset of putative assays based on their locus‐specific F_ST_ and position on reference scaffolds (to avoid selecting loci on the same scaffold). DAPC was used, as above, to ensure that this subset resulted in similar overall population structure and cluster separation, as compared to the full dataset. We then used samtools v1.8 (Li et al., 2009) to extract flanking sequences (100 bp in each direction) from the reference genome, and submitted these for assay design with rhAmp SNP genotyping technology (Integrated DNA Technologies, Coralville, IA).

From the loci for which rhAmp assays could be designed, we selected 96 assays to test using a Fluidigm Biomark 96.96 dynamic array (Fluidigm Corporation, South San Francisco, CA). We created positive controls for all assays by synthesizing gBlock synthetic Gene Fragments (Integrated DNA Technologies, Coralville, IA) representing either the reference or alternate alleles for each SNP. To create these positive controls, we extracted sequences from the reference genome (again, using samtools) which spanned the allele‐specific and locus‐specific primers of the rhAmp assays plus 3 bp and 10 bp on the 5' and 3' ends, respectively, of these fragments (to provide padding between assays as recommended by Integrated DNA Technologies). We then concatenated these fragments into total lengths of up to 1,000 bp (each containing positive control sequences for 7–11 assays). These fragments were synthesized for both the reference and alternate alleles for each SNP, and a heterozygote positive control was created by mixing the reference and alternate at equal molarity. For each of the positive control types (reference, alternate, and heterozygote), we combined all individual gBlock fragments, resulting in a single positive control sample per control type for all assays (e.g., reference allele controls for all assays combined in a single tube). We used three dilutions (1:1,000, 1:10,000, and 1:100,000, from a stock 10 ng/µl) of these positive controls to mimic potential variability in DNA quality/quantity in intercepted samples.

All sample DNA was normalized to 10 ng/µl, and we prepared sample master mixes containing 3.5 µl 2× rhAmp genotyping master mix, 0.35 µl 20× Fluidigm GT Sample loading reagent, 0.175 µl 40× rhAmp reporter mix, 0.03 µl ROX reference dye (Invitrogen, Carlsbad, CA), 0.945 µl dH2O, and 2 µl of either the DNA sample or the diluted gBlock (7 µl total). Assay master mixes contained 2.5 µl 2× Fluidigm assay loading reagent and 2.5 µl 20× rhAmp SNP assay, and we followed manufacturer recommendations to use the Fluidigm Integrated Fluidic Circuit (IFC) Controller HX to prime and load the IFC cartridge. Thermal cycling and imaging were conducted using the Fluidigm Biomark as follows: 95°C for 10 min and 40 cycles of (95°C for 15 s, 63°C for 30 s, 68°C for 30 s) with imaging conducted at the end of each cycle.

### 2.6 Diagnostic marker data processing and analysis

We used the Fluidigm SNP Genotyping Analysis software (Fluidigm Corporation, South San Francisco, CA) to process data from the Biomark and call genotypes. Genotypes were called at cycle 40 using the default confidence threshold of 65, NTC normalization, and K‐means clustering. The final genotyping was done in triplicate per each sample and control (on a single IFC cartridge) and included samples to validate the assays (identical DNA samples that were included in the ddRAD libraries) and test specimens with unknown origins and genotypes; we used different genotyping rules for validation versus test individuals. For validation individuals, we required two of the triplicates to agree on the ddRAD genotype to be considered correct validation, and two correct triplicates with high confidence (>95%) overruled one incorrect genotype. We only allowed a single triplicate to be considered a correct validation of the genotype when the other two triplicates did not result in a genotype call. For test individuals, we generally required triplicate genotypes to match in order to call a genotype for the specimen; if a specimen displayed any mismatch between triplicates, it was coded as missing data. The only exception to this rule was if triplicates failed (and did not result in a genotype call); in these cases, we called a genotype for the specimen if the confidence of the called genotype(s) was >95% or if separation between genotypes across the entire assay was very distinct, but the confidence call was between 80% and 95%.

We used DAPC to analyze the dataset resulting from the diagnostic panel. The main DAPC workflow was identical to that of the main dataset; however, the test specimens with unknown origins were not included in the main analysis and model construction, but instead were treated as supplemental individuals (Jombart & Collins, 2017). This process involves first conducting the main DAPC on the core dataset and then statistically transforming the supplemental individuals’ allele data with the centering and scaling of the initial DAPC model. The position of the supplemental individuals onto the original discriminant functions can then be predicted with the discriminant coefficients of the original model (Jombart & Collins, 2017). In this way, we can use the range‐wide sampling of *A. ludens* to predict the geographic origin of intercepted specimens and assign a confidence score to that prediction based on the fit of these supplemental individuals to the initial DAPC model. Our final dataset for this analysis consisted of ddRAD genotypes for all individuals in the main, filtered dataset, combined with Biomark genotypes for the test specimens.

## 3 RESULTS

### 3.1 *A. ludens* reference genome

To generate a reference genome for read mapping of ddRAD data, after filtering, 39.9 Gb of fragment library (~61.9× coverage) and 17.9 Gb of mate‐pair library data (~27.8× coverage) were used with ALLPATHS‐LG, which resulted in an assembly containing 152,464 contigs >1 kb (N50 = 6.1 kb) placed onto 44,974 scaffolds (N50 = 43 kb). The estimated genome size for this species, based on Kmer abundance of these data, is 644.7 Mb, and our assembly is 683.0 Mb in length with 20% of the genome as scaffold gaps. Overall the contiguity of this assembly was marginal, with most protein coding genes likely to span contigs and scaffolds. However, this is sufficient to use as a reference for ddRAD data mapping, as it allows higher confidence in generating catalog loci (in *Stacks*) versus de novo ddRAD data analysis methods which can be confounded by contamination and repetitive genomic sequences when not anchored to a reference.

### 3.2 ddRAD data processing

A total of 563 individuals (Table 1) and six negative control libraries were sequenced across three ddRAD libraries, resulting in 755 million reads (see Table S1 for detailed specimen information). Filtering with *process_radtags* yielded 725 million reads, and 637 million of these were successfully mapped to the *A. ludens* reference genome (an average of 1.1 million per specimen, Table S1). Initial data processing removed 195 individuals that had high missing data, and we removed an additional 12 individuals from Mexico with vague locality data, leading to a final dataset of 356 individuals and 2,081 SNPs (336 of these individuals were wild caught, and 20 from rearing strains) (Table 1).

**Table 1:** Descriptive population genetic statistics for regions (abbreviated) and state divisions, for the ddRAD and diagnostic SNP datasets

Population	*N*_RAW_	*N*_FIL_	2,081 ddRAD SNPs				28 diagnostic SNPs			
			*H*_O_	*H*_E_	*G*_IS_	Div	*H*_O_	*H*_E_	*G*_IS_	Div
W MEX	100	86	0.157	0.150	−0.052	0.149	0.154	0.177	0.129	0.173
E MEX/TX	293	159	0.154	0.146	−0.056	0.145	0.187	0.201	0.068	0.196
GTM/BEL/HON	50	34	0.155	0.147	−0.056	0.146	0.178	0.217	0.178	0.214
CR/PAN	88	57	0.147	0.139	−0.056	0.138	0.175	0.199	0.123	0.200
Texas	90	32	0.148	0.141	−0.052	0.139	0.179	0.208	0.142	0.203
Chiapas(M)	9	8	0.157	0.147	−0.068	0.145	0.156	0.214	0.270	0.205
Colima(M)	15	13	0.154	0.143	−0.071	0.142	0.168	0.177	0.053	0.168
Guerrero(M)	10	7	0.144	0.139	−0.042	0.136	0.164	0.207	0.205	0.207
Hidalgo(M)	18	13	0.161	0.147	−0.092	0.147	0.210	0.206	−0.016	0.205
Jalisco(M)	42	34	0.158	0.149	−0.061	0.148	0.147	0.163	0.096	0.159
México(M)	14	13	0.163	0.150	−0.084	0.149	0.187	0.190	0.020	0.187
Michoacán(M)	13	12	0.160	0.148	−0.080	0.147	0.134	0.171	0.217	0.167
Nayarit(M)	16	9	0.155	0.147	−0.053	0.146	0.136	0.175	0.223	0.177
Nuevo León(M)	78	35	0.156	0.147	−0.064	0.146	0.191	0.197	0.029	0.194
Querétaro(M)	20	17	0.154	0.147	−0.050	0.145	0.204	0.216	0.056	0.213
San Luis Potosí(M)	9	7	0.155	0.126	−0.230	0.123	0.177	0.178	0.007	0.159
Tamaulipas(M)	38	29	0.155	0.144	−0.079	0.142	0.168	0.179	0.061	0.177
Veracruz(M)	21	16	0.155	0.145	−0.073	0.143	0.221	0.187	−0.180	0.178
Guatemala	15	6	0.158	0.142	−0.117	0.139	0.180	0.177	−0.017	0.179
Belize	16	14	0.156	0.145	−0.076	0.144	0.179	0.188	0.045	0.186
Honduras	19	14	0.153	0.143	−0.073	0.141	0.181	0.227	0.202	0.221
Costa Rica	51	37	0.144	0.138	−0.046	0.136	0.162	0.185	0.124	0.188
Panama	37	20	0.153	0.138	−0.109	0.136	0.195	0.211	0.076	0.210
Family 10	7	7	0.121	0.105	−0.147	0.103	0.163	0.163	0.002	0.162
Tapachula 7	8	5	0.127	0.105	−0.205	0.103	0.155	0.205	0.243	0.205
Willacy	8	8	0.138	0.122	−0.128	0.121	0.165	0.162	−0.014	0.157

Note

Mexican states and rearing strains indicated with “(M)” and “(R)”, respectively.

Abbreviations: *N*_RAW_: original sample sizes per region/state division; *N*_FIL_: filtered sample sizes per region/state division; *H*_O_: observed heterozygosity; *H*_E_: expected heterozygosity; *G*_IS_: inbreeding coefficient; Div: gene diversity (1–*Q*_inter_); W MEX: west Mexico; E MEX/TX: east Mexico and Texas; GTM/BEL/HON: Guatemala, Belize, and Honduras; CR/PAN: Costa Rica and Panama.

### 3.3 Population genetic analyses

**Figure 1:**
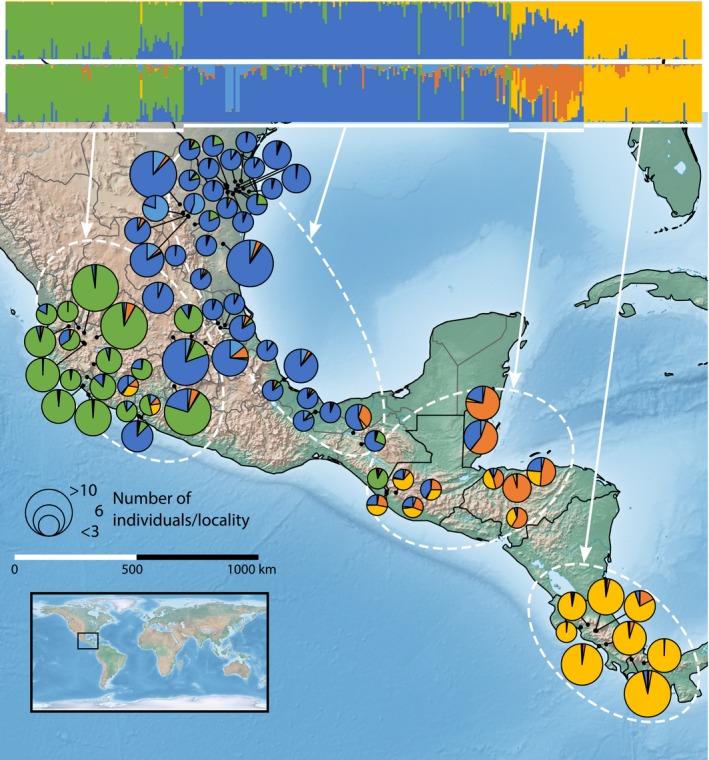
STRUCTURE barplot results for *K* = 3 (top bar plots, above) and *K* = 5 (top barplots, below), and *K* = 5 results displayed as average cluster membership per collection locality. Pie chart size indicates sample size, and some singleton localities in Texas with similar genetic cluster memberships have been combined. Individuals in STRUCTURE barplots are ordered generally by genetic cluster, then west to east by state (in Mexico) and country. White bars and arrows below STRUCTURE barplots correspond to the four main regions (see Results)

STRUCTURE analyses with varied parameters resulted in similar estimates of population structure for the supported values of *K* (see below). Analyses using default parameters and those with alternative ancestry prior and a specified α resulted in virtually identical results. Using a location prior identified the same main clusters, but tended to increase admixture in all individuals, and analyses including rearing strains consistently identified those individuals as a unique cluster (we focus on differentiation of rearing individuals with DAPC, below). Given the similarity of these analyses, we focused on and present the results of the analyses with default parameters, excluding the rearing strains (results for all analyses, as well as Ln Pr(*X|K*), *ΔK*, and the Puechmaille statistics, provided in Figures S1, and comparative summary of different analyses provided in Figure S2). We used multiple methods to determine the most likely value of *K*. At the broadest level, *K* = 3 is well supported by a clear peak in values of *ΔK* and a broad plateau in the values of Ln Pr(*X|K*). The Puechmaille statistics supported *K* = 5, 6, and 7, and Ln Pr(*X|K*) showed a second, finer plateau at around *K* = 7 and a peak at *K* = 10. Geographically, the *K* = 3 clusters corresponded to (a) west Mexico (west of the Mexican Plateau), (b) Texas and east Mexico, and (c) Costa Rica and Panama, with populations in Guatemala, Belize, and Honduras being intermediate to the latter two clusters (Figure 1). At *K* = 5, Guatemala, Belize, and Honduras were distinct, and some locality‐based substructure was apparent in Texas and east Mexico cluster. At both *K* = 3 and *K* = 5, there was admixture apparent in individuals from all genetic clusters (Figure 1). Above *K* = 5, the latter cluster was increasingly subdivided, but the resulting clusters did not correspond to collection localities. Given these results, we treated *K* = 5 as the best‐supported value of *K* using STRUCTURE.

**Figure 2:**
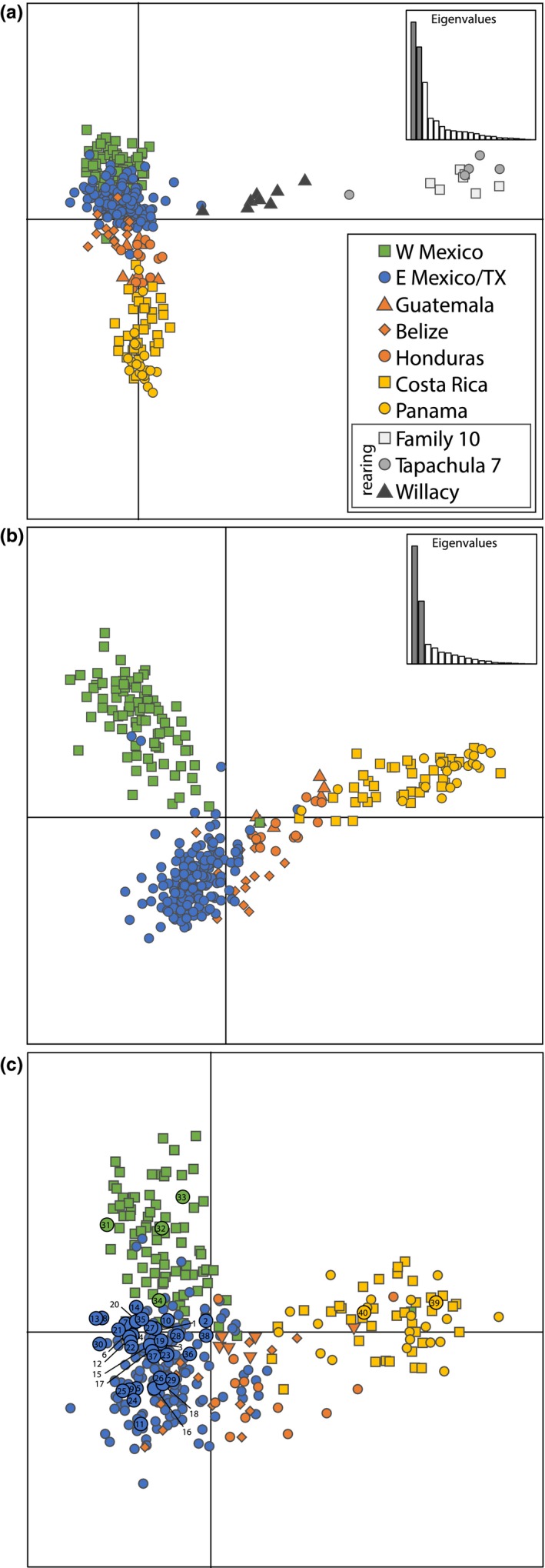
Results of discriminant analysis of principal components (DAPC) for ddRAD SNP dataset (2,081 SNPs) (a and b) and diagnostic SNP dataset (28 SNPs) (c). (a) shows analyses including individuals from rearing strains, and (b) shows analyses excluding those individuals. Individuals are colored roughly according to clusters in Figure 1. For the diagnostic SNP dataset (c), numbered circles with thick black outlines represent intercepted test individuals; colors within these shapes correspond to the pre‐existing cluster they were assigned to, and numbers correspond to specimen identifiers in Table 4 and Table S2. Inset graphs in a and b show relative explanatory power of eigenvalues in the analyses

Discriminant analysis of principal components resulted in similar population structure as STRUCTURE. The *find.clusters* algorithm predicted *K* = 4 for the analysis including rearing individuals, and *K* = 3 for the analysis excluding rearing individuals. When rearing individuals were included, all three rearing strains formed a single distinct cluster as predicted by *find.clusters* and were highly distinct compared to all of the wild individuals (Figure 2a). The Willacy strain was intermediate between the two strains maintained in Guatemala and the wild individuals from east Mexico and Texas, which is not surprising given that it was established from Wild flies collected in Raymondville, Texas, in 2008, and has been maintained in colony for approximately ~75 generations (H. Conway, *personal communication*). The other three clusters predicted by *find.clusters* matched those predicted when rearing strains were excluded, although with better separation in the latter, and corresponded to west Mexico, east Mexico and Texas, and Costa Rica/Panama (Figure 2b). Similar to the STRUCTURE results, there were individuals that were intermediate or mixed between these clusters, particularly those from the intergradation between east Mexico and isthmian Central America, as well as several individuals that did not conform to the same genetic cluster as other individuals collected in the same trap (e.g., three individuals from east Mexico localities (blue circles in Figure 2b) that clustered more west Mexico individuals (green squares in Figure 2b)). Pairwise F_ST_ between the four main clusters ranged from 0.021 (east Mexico/Texas versus Guatemala/Belize/Honduras) to 0.096 (west Mexico versus Costa Rica/Panama) (Table 2).

**Table 2:** Pairwise *F*_ST_ values for regional divisions

	W MEX	E MEX/TX	GTM/BEL/HON	CR/PAN
W MEX	–	0.239	0.324	0.441
E MEX/TX	0.047	–	0.063	0.302
GTM/BEL/HON	0.062	0.021	–	0.161
CR/PAN	0.096	0.057	0.040	–

Note

Numbers below the diagonal are for ddRAD dataset (2,081 SNPs), and those above the diagonal are for the diagnostic SNP dataset (28 SNPs). All comparisons were significant after Bonferroni correction.

Abbreviations: W MEX: west Mexico; E MEX/TX: east Mexico and Texas; GTM/BEL/HON: Guatemala, Belize, and Honduras; CR/PAN: Costa Rica and Panama.

Taken together, STRUCTURE and DAPC strongly support distinction between west Mexico, east Mexico/Texas, and isthmian Central America. The intermediate region of Guatemala, Belize, and Honduras is less genetically distinct, and intergradation is apparent between its neighboring clusters, but it is differentiated at a finer scale. We calculated basic population genetic statistics at two scales, one considering these four regions as populations, and one recognizing finer‐scale populations, including state divisions in Mexico and the United States and country divisions throughout Central America (hereafter referred to as “state divisions”). Although these finer‐scale boundaries are biologically arbitrary, they allow us to delineate localities while accommodating the fact that many of the sampling localities had small sample sizes (when comparing these state divisions to population clusters from STRUCTURE and DAPC, and ignoring minor admixture (*Q* < 0.4), only one state (Querétaro) contains individuals from both the west Mexico cluster and the east Mexico/Texas cluster, although the individuals belonging to each cluster were from separate collection localities). Descriptive population genetic statistics were quite similar when considering the regional divisions, and all individual measurements were relatively moderate. Observed heterozygosity and gene diversity ranged from 0.147 and 0.138, respectively, in Costa Rica and Panama to 0.157 and 0.149, respectively, in west Mexico (Table 1). At the finer scale of state divisions, we observed only slightly more variation in these statistics, with observed heterozygosity ranging from 0.144 in Costa Rica to 0.163 in the state of México and gene diversity ranging from 0.123 in the Mexican state of San Luis Potosí to 0.149 in the state of México. At both regional and state scales, G_IS_ indicated less relatedness between individuals in populations than would be expected with random mating. Finally, despite low sample sizes, the rearing strains exhibited characteristics expected from population bottlenecks (lower heterozygosity and gene diversity); however, G_IS_ was negative, as in the wild populations, indicating that inbreeding associated with mass rearing is not substantially increasing relatedness between individuals (Table 1). Overall, there were no patterns of considerably higher genetic diversity or heterozygosity in some geographic areas compared to others. Average pairwise F_ST_ between these population divisions was 0.084 and ranged from 0.006 (Nayarit versus Jalisco) to 0.266 (San Luis Potosí versus Tapachula 7) (Table 3).

**Table 3:** Pairwise *F*_ST_ values for state divisions

	(1)	(2)	(3)	(4)	(5)	(6)	(7)	(8)	(9)	(10)	(11)	(12)	(13)	(14)	(15)	(16)	(17)	(18)	(19)	(20)	(21)	(22)
(1) Texas	–	0.068	**0.223**	0.082	0.026	**0.281**	0.109	**0.285**	**0.230**	−0.008	0.012	0.017	0.002	0.009	0.138	0.050	**0.134**	**0.345**	**0.284**	**0.455**	**0.344**	**0.382**
(2) Chiapas (M)	**0.025**	–	0.237	−0.022	0.070	**0.318**	0.129	0.289	0.257	0.055	0.068	0.020	0.070	0.052	0.048	0.037	0.056	**0.222**	**0.198**	**0.505**	0.385	0.394
(3) Colima (M)	**0.070**	**0.061**	–	0.179	**0.309**	−0.009	0.073	0.028	−0.028	**0.231**	0.132	**0.311**	**0.291**	**0.189**	**0.271**	**0.328**	**0.377**	**0.471**	**0.415**	**0.448**	**0.339**	**0.276**
(4) Guerrero (M)	0.010	0.020	0.052	–	0.110	**0.224**	0.070	0.158	0.182	0.069	0.023	0.098	**0.147**	0.115	0.010	0.093	0.134	**0.266**	**0.213**	0.442	0.284	0.269
(5) Hidalgo (M)	**0.026**	0.029	**0.083**	0.030	–	**0.373**	**0.202**	**0.361**	**0.328**	0.018	0.069	0.031	0.017	**0.104**	**0.219**	0.040	**0.132**	**0.342**	**0.293**	**0.535**	**0.437**	**0.445**
(6) Jalisco (M)	**0.066**	**0.055**	**0.014**	**0.049**	**0.074**	–	0.102	0.012	−0.033	**0.284**	**0.185**	**0.369**	**0.349**	**0.251**	**0.300**	**0.396**	**0.436**	**0.503**	**0.463**	**0.418**	**0.334**	**0.268**
(7) México (M)	**0.046**	**0.033**	**0.036**	**0.032**	**0.053**	**0.029**	–	0.082	0.076	**0.119**	0.046	0.211	**0.174**	0.072	0.135	**0.230**	**0.261**	**0.390**	**0.329**	**0.395**	0.273	**0.242**
(8) Michoacán (M)	**0.064**	0.050	0.025	0.043	**0.072**	**0.015**	**0.030**	–	−0.002	**0.287**	0.181	**0.372**	**0.360**	**0.268**	0.265	**0.384**	**0.400**	**0.463**	**0.416**	**0.393**	0.292	**0.191**
(9) Nayarit (M)	**0.068**	**0.054**	0.019	0.045	**0.081**	0.006	**0.027**	0.017	–	**0.243**	0.142	0.323	**0.309**	0.208	0.254	**0.349**	**0.366**	**0.464**	**0.408**	**0.389**	0.279	0.274
(10) Nuevo León (M)	**0.011**	0.023	**0.066**	0.019	0.021	**0.064**	**0.042**	**0.060**	**0.061**	–	0.007	0.019	0.001	0.024	**0.139**	0.044	**0.136**	**0.327**	**0.273**	**0.481**	**0.372**	**0.397**
(11) Querétaro (M)	**0.013**	0.020	**0.039**	0.007	**0.025**	**0.035**	**0.023**	**0.033**	**0.035**	**0.017**	–	0.054	0.057	0.013	0.120	0.090	**0.164**	**0.340**	**0.278**	**0.420**	0.298	**0.298**
(12) San Luis Potosí (M)	**0.060**	**0.078**	**0.124**	0.083	**0.071**	**0.107**	**0.091**	**0.112**	**0.115**	**0.052**	**0.060**	–	0.035	0.056	0.211	0.030	0.122	**0.368**	**0.322**	0.570	0.442	0.478
(13) Tamaulipas (M)	**0.008**	0.025	**0.072**	0.019	**0.021**	**0.068**	**0.046**	**0.065**	**0.068**	**0.012**	**0.015**	**0.055**	–	0.038	**0.204**	0.037	**0.148**	**0.349**	**0.300**	**0.535**	**0.432**	**0.462**
(14) Veracruz (M)	**0.012**	0.019	**0.065**	0.018	0.021	**0.061**	**0.043**	**0.060**	**0.062**	0.013	0.016	**0.061**	0.012	–	**0.155**	0.086	**0.160**	**0.357**	**0.301**	**0.485**	**0.368**	**0.390**
(15) Guatemala	**0.034**	0.022	**0.076**	0.030	0.040	**0.070**	**0.047**	0.064	0.077	0.031	0.033	0.098	0.033	0.034	–	0.181	0.129	**0.198**	**0.154**	0.464	0.313	0.403
(16) Belize	**0.039**	0.037	**0.090**	0.049	**0.040**	**0.083**	**0.061**	**0.081**	**0.084**	**0.034**	**0.042**	**0.078**	**0.033**	**0.038**	0.040	–	0.071	**0.266**	**0.213**	**0.556**	**0.450**	**0.477**
(17) Honduras	**0.045**	**0.037**	**0.097**	**0.048**	**0.046**	**0.092**	**0.069**	**0.087**	**0.095**	**0.042**	**0.051**	**0.095**	**0.046**	**0.043**	0.030	**0.040**	–	**0.164**	**0.142**	**0.517**	**0.436**	**0.475**
(18) Costa Rica	**0.062**	**0.058**	**0.115**	**0.061**	**0.073**	**0.112**	**0.088**	**0.105**	**0.115**	**0.064**	**0.065**	**0.110**	**0.064**	**0.068**	0.037	**0.063**	**0.050**	–	0.036	**0.584**	**0.518**	**0.540**
(19) Panama	**0.081**	**0.075**	**0.126**	**0.082**	**0.084**	**0.121**	**0.100**	**0.120**	**0.125**	**0.077**	**0.082**	**0.119**	**0.079**	**0.081**	0.044	**0.075**	**0.064**	**0.028**	–	**0.547**	**0.463**	**0.506**
(20) Family 10	**0.165**	**0.188**	**0.181**	0.188	**0.188**	**0.156**	**0.159**	**0.168**	0.175	**0.170**	**0.159**	0.260	**0.176**	**0.186**	0.209	**0.204**	**0.210**	**0.212**	**0.234**	–	0.074	**0.353**
(21) Tapachula 7	**0.168**	0.179	**0.184**	0.194	**0.187**	**0.160**	**0.163**	**0.167**	0.181	**0.173**	**0.160**	0.267	**0.177**	**0.185**	0.206	**0.197**	0.210	**0.213**	**0.232**	0.096	–	0.284
(22) Willacy	**0.100**	**0.122**	**0.113**	0.109	**0.130**	**0.105**	**0.092**	**0.102**	**0.112**	**0.110**	**0.098**	**0.191**	**0.118**	**0.118**	0.138	**0.138**	**0.147**	**0.157**	**0.178**	**0.144**	0.156	–

Note

Numbers below the diagonal are for ddRAD dataset (2,081 SNPs), and those above the diagonal are for the diagnostic SNP dataset (28 SNPs). Bolded values indicate significant comparisons after Bonferroni correction, states in Mexico are denoted with “(M),” and numbers preceding population names correspond to column numbers.

### 3.4 Diagnostic panel design

To generate a diagnostic panel of markers from the ddRAD data, we submitted 118 SNP sites for assay design. After removing loci for which assays could not be designed, we filtered this set down to 96 assays to synthesize and test. We used DAPC to ensure that similar population separation was achieved with these 96 loci, compared to the ddRAD dataset. We tested the performance of these 96 assays by genotyping 96 individuals (including multiple positive controls, labeled “validation1” in Table S1) from the ddRAD data using the Fluidigm Biomark (a single technical replicate per individual). We used this run simply to determine whether assays worked across a range of specimens or failed (i.e., we did not verify ddRAD genotypes according to the rules described in the methods), and from these results picked 32 assays which showed the greatest qualitative success. Again, DAPC was used to verify population separation with these 32 SNPs.

Our rationale for choosing 32 assays for the diagnostic panel, from the original 96, was to allow the genotyping of 96 individuals in triplicate on a single 96.96 IFC cartridge on the Fluidigm Biomark (i.e., being able to run each assay three times, as technical replicates, on a single IFC cartridge). The 96 individuals we used for genotyping validation and assay testing included two sets of individuals from the ddRAD dataset which represented the fewest number of individuals with as many ddRAD genotypes as possible (labeled “validation1” and “validation2” in Table S1). These two sets theoretically provided 2 replicates of each genotype and amounted to 21 and 23 individuals. We then included 40 specimens, mostly of unknown origin, to act as a real‐world test of this diagnostic panel (Table 4, Table S2). This included flies and maggots collected as part of regular monitoring efforts in California and Texas, as well as seven and two specimens from Mexico and Panama, respectively, which were specimens with questionable origins as determined with mtDNA in Ruiz‐Arce et al. (2015). Finally, we included four reference, four alternate, and three heterozygote positive controls at varying concentrations, and a no‐template control to complete this 96‐specimen test dataset.

**Table 4:** Specimen information and diagnostic panel results for 40 test specimens

No.	Collection locality; trap/host; collection date [Ruiz‐Arce et al., 2015 haplotype and predicted origin]	W Mexico	E Mexico/TX	Guatemala	Belize	Honduras	Costa Rica	Panama
1	USA: TX, La Feria; MBT, grapefruit; 27.iii.2014	0.085	0.895*	0.019	0.001			
2	USA: TX, La Feria; MBT, grapefruit; 27.iii.2014	0.123	0.603*	0.178	0.001	0.096		
3	USA: TX, San Benito; MBT, orange; 28.iii.2014	0.036	0.957*	0.004	0.003	0.001		
4	USA: TX, San Benito; MBT, orange; 28.iii.2014	0.057	0.936*	0.006				
5	USA: TX, La Feria; MBT, grapefruit; 11.iv.2014	0.001	0.997*	0.001	0.001	0.000		
6	USA: TX, La Feria; MBT, grapefruit; 11.iv.2014	0.056	0.943*	0.001	0.001	0.000		
7	USA: TX, Weslaco; MBT, grapefruit; 7.iv.2014	0.132	0.867*	0.001				
8	USA: TX, Weslaco; MBT, grapefruit; 7.iv.2014	0.181	0.818*	0.001				
9	USA: TX, Lyford; MBT, sour orange; 17.iv.2014	0.001	0.997*	0.002				
10	USA: TX, Lyford; MBT, sour orange; 17.iv.2014	0.151	0.832*	0.015	0.002			
11	USA: TX, Weslaco; MBT, calamondin; 15.iv.2014	0.000	0.944*		0.051	0.005		
12	USA: TX, Weslaco; MBT, calamondin; 15.iv.2014	0.035	0.960*	0.005				
13	USA: TX, Weslaco; MBT, sour orange; 28.iv.2014	0.172	0.827*	0.001				
14	USA: TX, Weslaco; MBT, sour orange; 28.iv.2014	0.375	0.623*	0.001		0.001		
15	USA: TX, Brownsville; MBT, grapefruit; 27.v.2014	0.028	0.966*	0.004	0.001			
16	USA: TX, Edinburg; cut grapefruit; 10.vi.2015	0.002	0.933*		0.065			
17	USA: TX, Edinburg; cut grapefruit; 10.vi.2015	0.025	0.915*		0.059			
18	USA: TX, Edinburg; cut grapefruit; 10.vi.2015	0.002	0.914*		0.083			
19	USA: TX, Alamo; MBT, grapefruit; 4.iii.2016	0.047	0.945*	0.002	0.004	0.001		
20	USA: TX, Brownsville; MT, sour orange; 13.xi.2015	0.151	0.844*	0.004				
21	USA: TX, Brownsville; MYT, sour orange; 17.xii.2015	0.085	0.913*	0.002				
22	USA: TX, Brownsville; trap, sour orange; 7.i.2016	0.026	0.972*	0.002	0.001			
23	USA: TX, Laredo; orange; 4.viii.2016	0.016	0.979*		0.004			
24	USA: TX, Laredo; orange; 4.viii.2016	0.001	0.787*		0.211	0.001		
25	USA: TX, Laredo; sour orange; 4.viii.2016	0.002	0.964*		0.032	0.001		
26	USA: TX, Laredo; sour orange; 4.viii.2016	0.003	0.979*		0.017			
27	USA: TX, Laredo; sour orange; 4.viii.2016	0.122	0.868*	0.001	0.009			
28	USA: TX, Zapata; unknown; 23.ix.2016	0.080	0.836*	0.001	0.080	0.003		
29	USA: TX, Zapata; unknown; 23.ix.2016	0.003	0.627*		0.365	0.004		
30	USA: TX, Laredo; trap, citrus; 11.viii.2016	0.040	0.959*		0.001			
31	USA: CA, Spring Valley; MT, sapote; 6.xi.2017	0.996*	0.004					
32	Mexico: Nayarit, Jalisco; sour orange; 15.viii.2006 [AL03: inconclusive]	0.995*	0.005					
33	Mexico: Nayarit, Jalisco; sour orange; 15.viii.2006 [AL58: W Mexico]	1.000*						
34	Mexico: Michoacán, Nueva Italia; mango; 12.viii.2006 [AL32: Mexico]	0.513*	0.482	0.002	0.003	0.001		
35	Mexico: Michoacán, Nueva Italia; mango; 12.viii.2006 [AL03: inconclusive]	0.219	0.776*	0.001	0.003	0.001		
36	Mexico: Nuevo León, Allende; sour orange; 11.xi.2003 [AL03: inconclusive]	0.018	0.941*	0.003	0.035	0.002		
37	Mexico: Nuevo León, Allende; citrus trap; 19.xii.2002 [AL50: E Mexico]	0.017	0.940*	0.001	0.042			
38	Honduras: Morazan, Zamorano; sour orange; 15.ix.2004 [AL57: Honduras]	0.068	0.845*	0.009	0.013	0.065		
39	Panama: Chiriquí, Santa Clara; grapefruit; 29.v.2006 [AL03: inconclusive]						0.505*	0.495
40	Panama: Chiriquí, Santa Clara; grapefruit; 29.v.2006 [AL04: S Mexico/Central America]			0.013		0.007	0.552*	0.427

Note

Trap/host indicates either tree species in which a trap was hung (when trap type given), or host fruit that produced sample. “Ruiz‐Arce haplotype and predicted origin” refer to the results of Ruiz‐Arce et al. (2015). Asterisks indicate highest posterior probability of source prediction for each individual, and probability values < 0.001 have been removed. Detailed specimen information provided in Table S2.

Abbreviations: MBT: McPhail BioLure Trap; MYT: McPhail Torula Yeast Lure Trap; MT: McPhail trap (unknown bait).

### 3.5 Diagnostic panel genotyping

When considering all replicates of the 44 ddRAD validation specimens and the 40 test specimens (84 specimens × 96 assays (including replicates) = 8,064 potential genotypes), 6,138 of all potential genotypes (76.1%) resulted in a genotype call, and the average confidence score for successful genotypes was 97.3%. For the ddRAD validation individuals, there were 1,353 genotypes to validate (44 individuals × 32 loci, but with variable missing ddRAD genotypes); 1,041 of those were successfully validated (76.9%), 212 failed to amplify (no genotype call), and 100 resulted in failed validations (final genotype calls that differed from the ddRAD genotypes) (Table 5). Most of these failed validations (66/100) were caused by heterozygote ddRAD genotypes being called homozygotes for one allele or the other, which may be the result of poor‐quality input DNA and the stochastic nature of microfluidic reaction volumes (i.e., when DNA quality/quantity is low, the chance of individual DNA molecules making it into each microfluidic reaction chamber decreases). The possibility also exists that ddRAD genotypes were called incorrectly, but only 18 of the failed validations were for ddRAD genotypes with low (<10×) read depth. On a per‐locus basis, average genotyping success was 84.4% (minimum = 38.6%, maximum = 95.5%), and average validation success was 77.1% (minimum = 27.9%, maximum = 95.2%). Four of the 32 assays consistently failed or displayed unexpected results, so we excluded these assays from all further analyses. Our measures of success increased if these assays were excluded: 955 of 1,182 genotypes were successfully validated (80.7%), 161 failed to amplify, 66 resulted in failed validations, and per‐locus validation success averaged 80.9%; in summary, when excluding the four problematic assays and failed amplifications in the 28 locus diagnostic panel, we validated 93.5% of the ddRAD genotypes (955/(1,182–161)). Our positive controls worked well, as only 37 of 1,056 (3.5%) total positive control reactions failed, and different dilutions of each positive control produced consistent results. Qualitatively, these positive controls greatly increased our confidence in both automated genotype calls and manual editing of those calls.

**Table 5:** Diagnostic assay performance for validation of ddRAD genotypes, and test specimens

locus	ddRAD validated genotypes	ddRAD nonvalidated genotypes			ddRAD validation, other info		Test specimens			
		Fail	Miscall (high)	Miscall (low)	ddRAD het	ddRAD < 10	Genotyped	Geno < 3	Conflict	Miss
106_236865_4418	39/44 (0.89)	2	2	1	2	1	32/40 (0.80)	9	3	5
1097_90776_5934*	12/43 (0.28)	16	6	9	7	1	34/40 (0.85)	7	4	2
12014_11876_11681	24/44 (0.55)	18	0	2	2	0	21/40 (0.53)	9	1	18
1271_66264_14951	34/42 (0.81)	4	2	2	0	1	33/40 (0.83)	2	4	3
13195_13998_17239	34/38 (0.89)	4	0	0	0	0	34/40 (0.85)	9	3	3
13448_11843_18246	35/43 (0.81)	7	1	0	1	0	29/40 (0.73)	8	3	8
13906_6774_20306	40/44 (0.91)	3	0	1	1	0	39/40 (0.98)	13	1	0
1451_61933_23204*	17/44 (0.39)	27	0	0	0	0	22/40 (0.55)	7	0	18
146_152030_23838*	33/43 (0.77)	4	6	0	3	2	33/40 (0.83)	6	0	7
1585_55938_28568	35/41 (0.85)	5	1	0	1	0	30/40 (0.75)	16	1	9
169_46093_32582	33/42 (0.79)	3	3	3	1	1	30/40 (0.75)	10	3	7
169_115167_32520	34/43 (0.79)	8	0	1	1	0	30/40 (0.75)	8	1	9
169_157168_32535	35/40 (0.88)	4	0	1	1	1	34/40 (0.85)	15	1	5
1738_69256_34014	34/41 (0.83)	3	1	3	3	0	36/40 (0.90)	6	0	4
17836_10023_35444	38/42 (0.90)	4	0	0	2	0	32/40 (0.80)	9	4	4
18308_3413_37089	40/44 (0.91)	2	0	2	1	0	31/40 (0.78)	8	6	3
250_760_56581	40/42 (0.95)	2	0	0	0	0	37/40 (0.93)	10	0	3
2564_11816_57667	33/41 (0.80)	6	1	1	1	1	24/40 (0.60)	4	7	9
270_3398_60937	35/43 (0.81)	6	0	2	2	2	23/40 (0.58)	11	0	17
37767_810_80534	27/43 (0.63)	8	7	1	7	2	28/40 (0.70)	16	2	10
4501_1340_91074	34/44 (0.77)	5	1	4	5	1	33/40 (0.83)	17	1	6
4808_30565_94636	30/42 (0.71)	12	0	0	0	0	31/40 (0.78)	10	0	9
5231_23669_99330	36/42 (0.86)	4	1	1	0	0	33/40 (0.83)	11	6	1
528_23389_99792	36/42 (0.86)	4	1	1	2	1	32/40 (0.80)	12	1	7
62_334436_109976	38/43 (0.88)	4	1	0	1	0	25/40 (0.63)	12	9	6
636_114293_110548	27/43 (0.63)	12	3	1	4	1	28/40 (0.70)	9	0	12
64_189228_111723	31/42 (0.74)	10	1	0	1	0	31/40 (0.78)	12	2	7
70_287186_117070*	24/41 (0.59)	4	6	7	7	2	29/40 (0.73)	10	5	6
729_2947_118563	27/40 (0.68)	6	6	1	6	1	19/40 (0.48)	9	1	20
770_72175_121997	37/41 (0.90)	3	1	0	1	0	34/40 (0.85)	13	0	6
908_61119_132233	34/43 (0.79)	9	0	0	0	0	35/40 (0.88)	6	1	4
952_3897_135282	35/43 (0.81)	3	4	1	3	0	36/40 (0.90)	6	4	0
Total		212	55	45	66	18		310	74	228

Note

For ddRAD validation, failed validation (“fail”) indicates assay failure (no returned genotypes) and miscall indicates incorrect genotype call with either high or low confidence (“miscall (high)” and “miscall (low)”, respectively). The number of validated ddRAD genotypes plus all nonvalidated genotypes equals the total number of possible genotypes for validation (denominator values in “ddRAD validated genotypes”). “ddRAD validation, other info” indicates heterozygous ddRAD genotypes called as homozygotes (“ddRAD het”) and miscalled genotypes where ddRAD genotypes had < 10 reads (“ddRAD < 10”). For test specimens, columns indicate the total number of: successful genotypes called from < 3 technical replicates (“geno < 3”), failed (coded as missing) genotypes due to conflict between technical replicates (“conflict”), and failed genotypes due to missing data (i.e., failed assay reactions, “miss”). Asterisks indicate four loci removed from analyses.

For the 40 test specimens of unknown origin, there were 1,280 potential genotypes. A total of 228 genotypes failed to amplify, and 74 displayed variable genotype calls between replicates and were treated as missing data. This equated to 978 successful genotype calls, or a 76.4% successful call rate. Excluding the four assays that consistently failed or displayed unexpected results increased this only slightly to 76.8% successful call rate (860/1120 potential genotype calls) (Table 4). Taken together, the genotyping results of our diagnostic panel strongly support the use of both repeated genotyping (triplicate genotypes per individual) and the use of positive control measures when using SNP genotyping assays for such an approach, particularly when input DNA quality is not of consistently high quality.

### 3.6 Diagnostic panel analysis

The subset of 28 loci that made up the diagnostic panel were highly differentiated (Tables 2 and 3) and were able to roughly recreate the main population structure predicted with 2,081 SNPs (Figure 2a and b). Some loss of resolution and general “spreading” of clusters was observed, particularly in areas with admixture or intergradation between main clusters. In many cases where admixture or intergradation between clusters was present, probability values for population assignment were very similar for multiple a priori groups. For example, test specimen 40 was predicted to belong to the Costa Rica group (Table 4); however, its probabilities of assignment to Costa Rica and Panama were 0.505 and 0.495, respectively, which reflects that individuals from these two countries are genetically indistinguishable and belong to the same genetic cluster. In general, predictions were biologically sensible. For example, most specimens collected from Texas matched the east Mexico/Texas cluster, while specimen 31, collected in California, matched the west Mexico cluster (Figure 2c). For the specimens that we included from Ruiz‐Arce et al. (2015), this SNP panel both confirms and provided higher resolution for source determination as compared to mtDNA (Table 4).

## 4 DISCUSSION

Here, we used a combination of genomic strategies to assess population structure and develop pathway analysis tools in *A. ludens*. We first generated a reference assembly for this species, and while it was sufficient for mapping reads from the ddRAD dataset, the overall quality was poor in terms of being a reference for gene annotation and assessing genomic structure (we have ongoing efforts to generate a high‐quality reference for *A. ludens*). We then used ddRAD to assess the genomic population structure across *A. ludens’* range and designed and validated a set of cost‐effective SNP genotyping assays for source determination of intercepted specimens. From our population genomic dataset of 2,081 SNPs, we strategically subsampled markers to arrive at a panel of 28 SNPs capable of recreating the same broad population structure as the genomewide dataset. We validated this panel by genotyping specimens included in the ddRAD dataset and then genotyping a set of real‐world test specimens that were intercepted as part of regular surveillance efforts in California and Texas. This is the first genomic‐scale assay for source determination in a pest tephritid. It will serve as a proof of concept for other recurrently invading pest species and be used as a tool to support decision making in the management of *A. ludens*.

### 4.1 Patterns of population structure

We found strong support for three main genetic clusters, corresponding to west Mexico, east Mexico/Texas, and isthmian Central America, and at a finer‐scale, we observed a broad zone of intergradation and unique signatures in Guatemala, Belize, and Honduras. Differentiation between these clusters was relatively low‐moderate (maximum *F*_ST_ between main clusters = 0.096; Table 2), and putatively admixed individuals were observed in all clusters (discussed below). Compared to previous population genetic studies, this degree of population structuring is quite high. Using two mitochondrial genes, Ruiz‐Arce et al. (2015) found some support for genetic structure between the northern and southern parts of *A. ludens*’ range, corresponding to the Isthmus of Tehuantepec (the narrowest point of Mexico). However, this pattern was driven by the distribution of low‐frequency haplotypes, and the most prevalent haplotype (found in 65% of individuals) was geographically widespread. While we do observe some differentiation between the northern and southern parts of the distribution (comparing Mexico to Costa Rica/Panama), similar to that identified by Ruiz‐Arce et al. (2015), the large zone of intergradation in Central America fails to support a strong north–south divide for this species.

Some of the population structure observed in this dataset corresponds to biogeographical zones in Mexico and Central America (see Morrone, 2014). For example, the west Mexico cluster is bounded by the southwestern and western extents of the Sierra Madre Occidental and Sierra Nevada mountain ranges, respectively. Additionally, the faunal distinctiveness and South American affinity of Costa Rica/Panama have been historically recognized (Halffter, 1987) and are supported by this dataset. However, a unique difficulty in assessing the mechanisms and patterns of population structure in fruit flies is that anthropogenic movement of eggs or maggots in host fruit can be widespread Boontop, Schutze, Clarke, Cameron, & Krosch, 2017; Boykin, Shatters, Hall, Dean, & Beerli, 2010; Kendra, Hennessey, Montgomery, Jones, & Epsky, 2007; Shi, Kerdelhue, & Ye, 2012). Ruiz‐Arce et al. (2015) recognized this in their sampling of *A. ludens* with individuals collected in the region of a fruit packing house near Nueva Italia, Michoacan (sampling locality MX20 in Ruiz‐Arce et al. (2015)); these individuals showed widespread haplotype diversity and likely represented multiple geographic sources (Ruiz‐Arce et al., 2015). We included specimens from this same locality and found similar genetic affinities to geographically distant genetic clusters (e.g., the one individual in the west Mexico clade with *Q* = ~0.4 matching the Costa Rica/Panama cluster, Figure 1), thereby confirming the conclusions of Ruiz‐Arce et al. (2015).

Previous studies have come to contrasting conclusions regarding the ancestral range or geographic origin of *A. ludens*. Historically, northeastern Mexico was considered the ancestral range of the species, based on high levels of infestation in sapote (*Casimiroa *spp.: Rutaceae), a native genus to Mexico and Central America, and a diverse assemblage of parasitoids compared to other regions (Baker, Stone, Plummer, & McPhail, 1944). In contrast, Ruiz‐Arce et al. (2015) found higher haplotype diversity of mtDNA markers in Costa Rica/Panama and suggested Central America as the origin of *A. ludens*. Although we observed slightly higher pairwise differentiation when comparing Costa Rica/Panama to other regions, no measures of genetic diversity were substantially higher in this region. Furthermore, none of the population units that we recognized had substantially higher diversity or heterozygosity, which may be expected from the source of a population expansion (Boehm, Waldman, Robinson, & Hickerson, 2015; Comes & Kadereit, 1998; Taberlet, Fumagalli, Wust‐Saucy, & Cosson, 1998). The uniformity of genetic diversity across this distribution could be the result of multiple factors. Individual *A. ludens* have been known to commonly travel 5–8 km in their lifetime (and up to 37 km, Shaw, Sanchez‐Riviello, Spishakoff, Trujillo, & Loppez, 1967), and combined with the aforementioned anthropogenic movement, high connectivity and gene flow across the distribution are unsurprising. Although our population genomic dataset provides unprecedented resolution of the population structure of *A. ludens*, explicit biogeographical analyses will likely be required to identify its likely ancestral range, which is beyond the scope of the current study.

We included rearing strains of *A. ludens* to provide some context to the population structure of wild populations, and all rearing strains were quite divergent from the wild populations (Figure 2a). The Willacy strain was more genetically similar to the east Mexico/Texas cluster of wild populations, which is biologically reasonable, as it was started from a population in south Texas much more recently than the established genetic sexing strains of Tapachula 7 and Family 10 (which originated from a population in southern Mexico). Interestingly, all rearing strains shared some genetic characteristics (e.g., along the second axis of variation (the *y*‐axis) in Figure 2a or in STRUCTURE analysis, Figure S1d). While rearing lines are typically thought to be highly bottlenecked, it can still be expected that these lines will continue to evolve over time and may share some artificially selected traits due to common artificial rearing conditions. Research into the effect of genetic markers associated with artificial rearing would be valuable but would require far greater sampling of these lines than the present study (and preferably from multiple time points in the establishment of a rearing strain).

### 4.2 Diagnostic panels and performance

From a genomewide dataset of 2,081 SNPs, our diagnostic panel of 28 highly differentiated loci was capable of roughly reconciling the sampled genomic population structure of *A. ludens* and predicting the geographic source of 40 test specimens. These test specimens included both real‐world unknown specimens captured in regular monitoring efforts in Texas and California, and specimens with questionable origins as determined with mtDNA in Ruiz‐Arce et al. (2015). The source predictions resulting from our diagnostic panel were, biologically speaking, sensible, as the majority of the test specimens were intercepted in southern Texas and matched to the east Mexico/Texas cluster. Creating a diagnostic tool such as this one will inherently be limited by the population structure present in the system, and ultimately, in the realm of regulatory agencies and recurrently invading pest species, exclusion of populations as the potential source of intercepted material is often more obtainable and preferred than pinpointing the exact source (Barr et al., 2014). In this light, and particularly in the cases of tephritids which often exhibit weak population structure (as is the case here), this approach provides a cost‐effective tool for conducting such pathway analysis for recurrently invading pest species. Additionally, by using a multivariate analysis framework such as the one used here, this approach is amenable to automated data analysis through a tailored web portal (e.g., mvMapper: Dupuis, Bremer, Jombart, Sim, & Geib, 2017), which would increase its functionality for regulatory agencies and diagnostics laboratories.

Assessing the “success” or statistical power of such a diagnostic panel is not necessarily a straightforward task. Cross‐validation strategies (jackknifing or leave‐one‐out analysis) are often used to assess the success of a subset of loci for assigning individuals to particular populations. However, these methods have also been criticized for “high‐grading bias” stemming from the indiscriminate use of individuals for both constructing the model and evaluating its success (Anderson, 2010; Waples, 2010). Limited sampling per population (which thus limits potential for cross‐validation) and low differentiation between populations increase potential biases in these calculations of success/power (Waples, 2010). Both of these phenomena apply to the current datasets, so we are reticent to employ cross‐validation simply as a means to calculating some value of “success” for this diagnostic panel. Rather, we rely on our comprehensive approach to assessing the population genomic patterns in this system and focus on the broad concordance between results of the full dataset and the diagnostic panel (Figure 2).

Validation of ddRAD genotypes using rhAMP assays for the 28 loci was 80.9% successful when both failed validations and failed amplifications were included, and 93.5% successful when ignoring the failed amplifications. Given that the ddRAD dataset also includes missing genotypes means that these validation “success rates” are inherently dependent on the efficacy of the ddRAD dataset, as well as that of the rhAMP assays. In the case of our assays, most of the failed validations were due to heterozygotes (from their ddRAD genotype) being called as homozygotes with the rhAMP assay. When using a microfluidic genotyping platform, this is indicative of poor or inconsistent DNA quality/quantity, as it is less likely that a full set of template DNA molecules end up in each microfluidic reaction chamber (i.e., for a heterozygote individual, template for only one allele may make it into the reaction chamber). Although higher success would obviously be preferred in such a regulatory context, we feel that this level of validation is satisfactory given the inherent stochasticity of missing data when using both ddRAD and the Fluidigm Biomark. Including more loci and filtering loci based on genotype validation success (rather than our more qualitative filtering strategy to go from 96 assays to 28 assays) would potentially increase validation success rates if that is important to regulatory agencies.

Broccanello et al. (2018) had high success using rhAMP assays (compared to other assay types) with variable quantities of input DNA (0.1–100 ng). However, the input tissue in their study was of consistent high quality (0.2 g of fresh leaf tissue from cultivated sugar beet plants). In our case, many specimens were collected in extensive trapping networks, where several days may pass in between collections of specimens from traps. Thus, specimens may have been dead and exposed to high temperature and humidity conditions for multiple days before being transferred to ethanol, leading to highly variable DNA quality. While this characteristic is not ideal from a molecular biology perspective, it does provide a realistic context for how intercepted material is often collected and is realistic to how this tool will be implemented. Overall, the success of this diagnostic assay for our real‐world test specimens speaks to the utility of this approach despite potentially low‐quality input DNA. Furthermore, the variability in genotype validation speaks to the need for duplicate or triplicate genotyping per individual, as we conducted here; without this added effort, we would have been much less confident in the results of this diagnostic assay.

The cost of this approach is entirely dependent on the scale of the genotyping in question. A back‐of‐the‐envelope calculation of the cost of this panel of 28 loci run in triplicate on the BioMark platform, for a diagnostic laboratory that would be conducting hundreds to thousands of these assays (at current market price), is approximately $13 USD per individual (without DNA extraction or the cost of technician/scientist time). In a direct comparison, Broccanello et al. (2018) demonstrated that rhAMP assays were less expensive, faster to run, and more robust than comparable kits, although they used standard real‐time qPCR instruments to conduct the genotyping reactions. One of the major strengths of our approach from a regulatory context is that 96 individuals can be genotyped at that price (again, for up to 32 loci in triplicate) in ~3 hr using a Fluidigm BioMark platform with minimal time at the bench. Combined with user‐friendly data analysis using the Fluidigm SNP Genotyping Analysis software means that this approach could be implemented relatively easily at a regulatory diagnostics laboratory. This study lays a valuable foundation for the use of such diagnostic assays and will undoubtedly aid in future testing and validation for developing best practices (e.g., balancing cost with the number of loci and number of replicates required for a genotype call) in an official regulatory program.

## CONFLICT OF INTEREST

None declared.

## Data Availability

Raw sequencing data are available on the US National Center for Biotechnology Information, BioProjects PRJNA503495 (genome assembly) and PRJNA503512 (ddRAD). Data input files are available in the Dryad repository, https://doi.org/10.5061/dryad.3476709; Dupuis, Ruiz‐Arce, Barr, Thomas, and Geib (2019).
