# Supplementary material for: Range‐wide population genomics of the Mexican fruit fly: Toward development of pathway analysis tools
Source: Evol Appl. 2019 Jun 13;12(8):1641–60. doi: 10.1111/eva.12824 (PMC6708432; doi:10.1111/eva.12824)

Figure S1. All STRUCTURE results

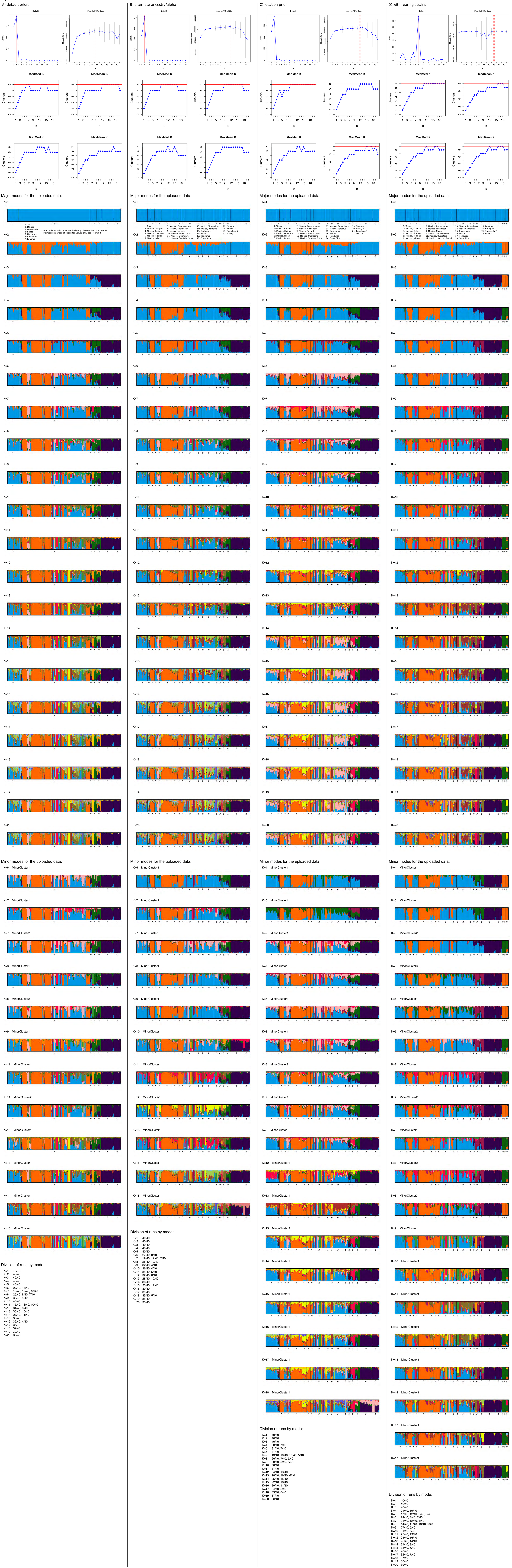

Supplement: Supplementary file 1 [file EVA-12-1641-s001.pdf]
