# Supplementary material for: Range‐wide population genomics of the Mexican fruit fly: Toward development of pathway analysis tools
Source: Evol Appl. 2019 Jun 13;12(8):1641–60. doi: 10.1111/eva.12824 (PMC6708432; doi:10.1111/eva.12824)

Figure S2. Comparinson of K = 5 STRUCTURE results with different priors (order of individuals as in Figure 1).

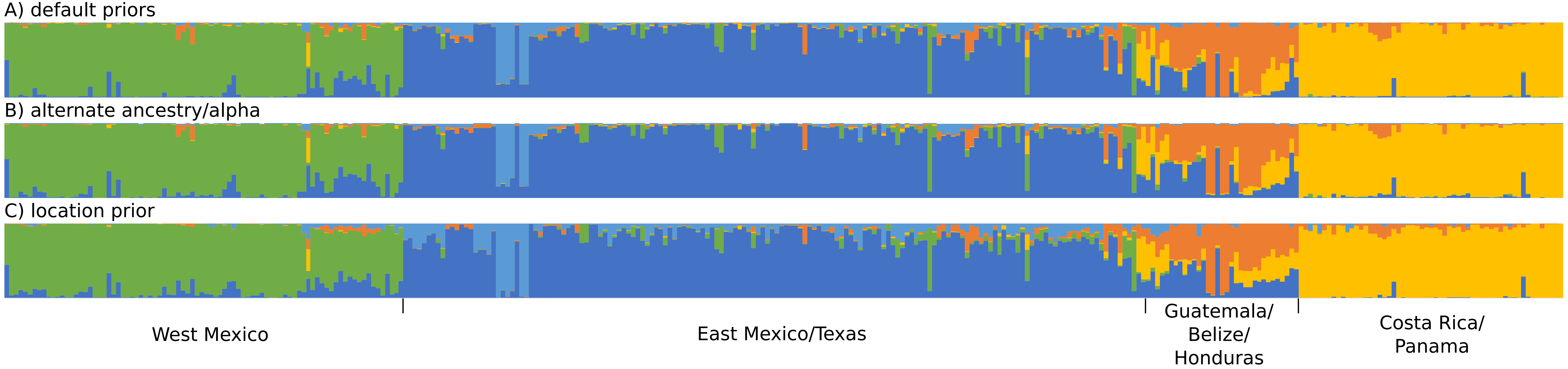

Supplement: Supplementary file 2 [file EVA-12-1641-s002.pdf]
